# Caregiver-centred empowerment for families raising autistic children: A qualitative case study from Argentina

**DOI:** 10.1177/13623613241238254

**Published:** 2024-03-18

**Authors:** Zsófia Szlamka, Cukier Sebastián, Charlotte Hanlon, Rosa A Hoekstra

**Affiliations:** 1 King’s College London, UK; 2 The London School of Economics and Political Science, UK; 3 Adolescentes y Adultos con Condiciones del Espectro Autista, Argentina; 4 Pedro de Elizalde Pediatric Hospital, Argentina; 5 Addis Ababa University, Ethiopia

**Keywords:** advocacy, Argentina, autism spectrum disorder, developmental disabilities, empowerment

## Abstract

Autistic children and their caregivers in Argentina often lack access to information, resources and evidence-based interventions. Caregiver empowerment may help support families to uphold their child’s rights and access suitable education and support. This study aimed to examine the perceptions on empowerment of caregivers of autistic children in Argentina. This was a phenomenological, qualitative study. We conducted 32 semi-structured individual interviews remotely. Participants included caregivers, health service providers, non-governmental organisation representatives, special education teachers and policy representatives. Data were analysed thematically. We developed three main themes: Caregiver agency: from intuitive coping strategies to entrepreneurship; ‘I had to cut down on therapy’: Economic instability and inequality affecting service access; and Equipping caregivers to be empowered. Both caregivers and professionals talked about the contribution of socio-economic inequalities to caregivers’ sense of disempowerment. Caregivers identified coping strategies and discussed their experiences with advocacy. They expressed that in-person and online support groups have an empowering effect. Based on participant views, strategies supporting caregiver empowerment may involve: interventions are co-designed by professionals and caregivers; focusing on caregiver mental health; and addressing the profound impact of poverty on the quality of life of families.

**Lay abstract:**

Caregivers of children with developmental disabilities, including autism, often struggle to access services, information and resources in Argentina. Little is known about how caregivers can be empowered to support their children as they wish to in the Argentinian setting. We spoke with 32 people online to understand existing and potential practices of supporting caregivers. The people we spoke with included caregivers, health service providers, non-governmental organisations’ representatives providing services or technical support, special education teachers and policy representatives. Participants said that poverty, and inequalities in accessing support, impact how caregivers can support their children. They mentioned examples that help caregivers feel empowered, such as peer support groups and caregiver training. Many caregivers spoke about how they became advocates for their children and how they developed initiatives such as advocacy campaigns and well-being support groups. Caregivers in Argentina may be empowered in various ways, and the following strategies can improve empowerment: strengthening collaboration between professionals and caregivers; focusing on caregiver mental health; and addressing the profound impact of poverty on the quality of life of families.

## Introduction

Many autistic children in South America receive no or insufficient support (Montiel-Nava et al., 2020). Service access across the region is influenced by lack of information, long waitlists in local service providers and high out-of-pocket costs (Montenegro et al., 2022). Autism remains highly stigmatised, and this stigma negatively impacts families’ access to services (Montenegro et al., 2022). Across the region, those who have access to private healthcare receive diagnoses at an earlier age than families depending on public health coverage (Montiel-Nava et al., 2024). In Argentina, financial difficulties and stark health inequalities prevent many families from accessing health care for their child (Paula et al., 2020). Approximately 36% of the Argentinian population has no formal health coverage (Palacios et al., 2020), and public health expenditure and the availability of health care staff are highly unequally distributed (Palacios et al., 2020). Around 40% of Argentina’s population and most of its economic activity are concentrated in the Buenos Aires Metropolitan Area (Muzzini et al., 2016). Meanwhile, the Northern Provinces of Argentina, such as Salta and Jujuy, suffer from poverty and lower living standards (Lozano Gracia et al., 2020), with 49% of households in Salta lacking basic needs (Lozano Gracia et al., 2020). Argentina’s high inflation rates further impact existing economic and health inequalities (Libman & Palazzo, 2020). Families’ service access is further limited by the fact that most services and autism specialists are located in the capital city, Buenos Aires. This means that those living in rural areas are facing difficulties accessing services at all (Moldavsky et al., 2011). Moreover, health and education professionals often need additional training regarding autism (Kowalczuk, 2021). The global COVID-19 pandemic further challenged service access for autistic people in Argentina, because families with no access to technology or stable Internet could not benefit from remote support (Valdez et al., 2021).

Despite the challenges, there are ongoing efforts to improve support systems for families. The age of diagnosis for autism has been decreasing, while awareness and services available have been increasing (Montiel-Nava et al., 2020). Autistic children in Argentina are often offered psychodynamic therapy and have access to speech, occupational and behavioural therapy – although as children reach adolescence, the number of hours of support they receive decreases substantially (Paula et al., 2020). Argentina is one of the few countries in Latin America with a solid legal framework protecting autistic individuals (Montiel-Nava et al., 2020). This framework includes a law that covers most health services for people with disabilities (Argentine Ministry of Health and Social Development, 1997, 2019) and a unified disability certificate allowing access to clinical and educational services (Argentine Ministry of Health and Social Development, 2001). Research and advocacy initiatives to support autistic people and their families are increasing thanks to organisations such as the Latin American Autism Network (Comunidad de Estados Latinoamericanos, 2013), where Argentinian members are active participants. One line of research focuses on the adaptation and implementation of community-based techniques and caregiver-mediated interventions for families (Rattazzi, 2014). Furthermore, screening tools and interventions are being adapted to the local context (Cuesta-Gómez et al., 2016; Rattazzi, 2014). Local inclusive education initiatives exist that aim to improve teacher training and educational tools (Valdez, 2009; Valdez et al., 2016).

Existing literature on family support identifies caregiver empowerment as a strategy to increase the capacity of families with an autistic child to support their children in ways they wish to (Heflinger et al., 1997; Puttahraksa et al., 2021; Zuurmond et al., 2020). While the definitions of empowerment may vary, scholars typically associate the concept with shifting decision-making and power over resources to individuals, communities and organisations (Babatunde et al., 2022; Batliwala, 2007; Wallerstein & Bernstein, 1994). Empowerment is often categorised as psychological, focusing on individual skills and self-confidence (Cattaneo & Chapman, 2010); economic: highlighting the eradication of poverty and socio-economic inequalities (Jiménez-Solomon et al., 2016); or community-based, focusing on power and control within communities (Babatunde et al., 2022). Caregiver empowerment is associated with positive parenting outcomes and can be an important tool to help families cope with challenges (Weiss et al., 2015). In high-income settings, empowerment has been linked with higher self-esteem and the feeling that caregivers can contribute to their child’s development (Nachshen, 2005). In contrast, low levels of empowerment were found to be associated with low levels of service usage, low self-efficacy among caregivers and lower awareness of available social support (Wakimizu et al., 2011). Attending interventions informing caregivers about autism and support is associated with the feeling of being empowered (Heflinger et al., 1997). In Latin America, there is some evidence investigating the effects of caregiver empowerment. For example, in Colombia, caregiver empowerment was found to be an effective tool to help single mothers become active agents in supporting their children, in the context of the country’s colonial past and professional-caregiver relationships that tend to be paternalistic (Magaña et al., 2019).

### The current study

The aim of this study was to examine the perceptions of caregiver empowerment in the Argentinian context. We studied the following questions:

What are the perceptions of stakeholder groups involved in service development for autistic children about caregiver empowerment in Argentina?

What efforts are currently employed to address systemic social, economic, and educational barriers to accessing resources, information, and support services in Argentina?

What is the potential of interventions targeting caregivers of autistic children to empower families and their communities to overcome structural barriers to participation in society in Argentina?

We focused the study on families with autistic children in childhood age. We made this decision as we hoped to understand how empowerment can support families at a time when it is the caregiver who is likely the advocate for their child to access support and services.

## Methods

This was a qualitative, phenomenological study, using semi-structured interviews (Smith, 2008). Ahead of data collection, the first author spent 2 months in Argentina to familiarise herself with the context and build rapport with gatekeepers and potential participants. While initially interviews were scheduled to take place in person, all were conducted online to comply with COVID-19 restrictions.

### Participants

The following stakeholder groups were invited to participate: (a) caregivers of autistic children; (b) health service providers working with children affected by all types of developmental disabilities including autism; (c) representatives of non-governmental organisations (NGOs) providing services or technical support in the field of autism; (d) teachers in special education teaching; and (e) policy representatives working with autism. We recruited participants from a range of stakeholder groups involved in supporting autistic children to understand caregiver empowerment as a phenomenon. We aimed to take a rights-based and family-centred approach in this study. As we report findings, we place caregiver voices at the centre and compare other viewpoints against theirs. Furthermore, in economically under-resourced settings, the World Health Organization (WHO) recommends an inclusive response to programmes for children with developmental disabilities, not distinguishing between specific diagnoses. As well as being pragmatic, this approach may be less stigmatising. Therefore, it is relevant to consider the perspectives of caregivers whose children have different diagnoses and are of different age.

We aimed to purposively sample informants from urban, rural and remote areas across the country to include the voices of culturally different communities. Therefore, participants were invited from the following provinces: Buenos Aires, Tierra del Fuego, Rio Negro, La Rioja, Santa Cruz, Salta and Jujuy.

We recruited caregivers through caregiver training programmes that the Programa Argentino para Niños, Adolescentes y Adultos con Condiciones del Espectro Autista (PANAACEA) organised between October 2020 and July 2021. Other caregivers were recruited through caregiver organisations in the Province of Buenos Aires. A snowballing method was used to contact further organisations across the country (Noy, 2008).

#### Interview guides

Interviews lasted between 45 and 60 min. The interview guides were constructed based on previous literature on caregiver empowerment for (Puttahraksa et al., 2021; Zuurmond et al., 2020) and on caregiver experiences in Argentina (Kowalczuk, 2021; Rattazzi et al., 2016). We sought the feedback of caregivers and professionals who expressed an interest in participation about the interview guides. The following topics were covered with all participants: family needs, stigma, information availability about rights, caregiver empowerment and the perceptions of existing services. The phrasing differed across stakeholder groups. For caregivers, we avoided using technical terms such as *empowerment*. We operationalised empowerment in questions such as:

- Can you tell me about things that you find important to provide your child or family with?
- Is there something you would like to be able to provide but you cannot as for now?
- What would you like to provide to your child that you think would help your family?

With professionals we used more technical terms such as ‘empowerment’, ‘caregiver intervention’ and ‘evidence-base’. After each interview, the topic guide was revised to add new topics that participants raised. An example of such an addition was conflicts with health insurance providers. All interviews were conducted by the first author in Spanish. Field notes were recorded to support triangulation of data (Flick, 2004). For the full topic guides, see Supplementary file 1.

### Analysis

All interviews were transcribed and translated to English by a local research assistant. The first author organised an introductory training session about autism with the research assistant. The research assistant was in regular touch with the first author to clarify any potentially unclear components in the transcripts. The first and second authors checked the English translations against the Spanish originals to validate the translations. Data were analysed using reflexive thematic analysis and inductive coding: transcripts were coded into themes and subthemes (Braun & Clarke, 2021). We decided to use multiple coders as the first author is not from Argentina and may have otherwise misinterpreted the data without sufficient contextual knowledge. The number of themes was not pre-specified. Three themes were found to capture the findings relating to the research questions with sufficient depth.

The first author coded all interviews using an inductive approach. The second author, who is from Argentina, coded six interviews independently. The first and second authors met to discuss the initial codebook following their double-coded transcripts and then met regularly to develop the final codebook upon coding of all transcripts (see Supplementary file 2).

### Positionality

The first author’s views on empowerment were shaped by previous studies she had conducted in the field. She therefore assumed that caregiver empowerment is a helpful concept to explore in the Argentinian setting. When analysing data, this meant that the first author was looking primarily for the advantages of an empowerment approach. The first author was often referred to in her position as a foreign researcher. Many participants asked her about how her experiences with supporting caregivers in her country compare to theirs – initiating a dialogue as part of the research process.

### Community involvement

We collaborated with local caregiver and professional organisations. All caregiver and professional participants were invited to be involved in designing the research questions, interview guides, the analysis and interpretation of data. Most participants gave their feedback on at least one of these aspects as part of stakeholder consultation meetings. Upon completion of the initial data analysis, the first author visited Argentina and sought participants’ feedback after presenting a summary of the draft findings during three in-person presentations. Participant feedback contributed to the interpretation of themes. For example, participants highlighted the importance of existing caregiver-centred initiatives. The first author subsequently presented the final results in person to both caregiver and professional participants as part of caregiver and clinician meetings.

## Results

Overall, 32 individual interviews were conducted. Participants included caregivers (*n* = 15); clinicians (*n* = 9); NGO representatives (*n* = 3); policy representatives (*n* = 2); and teachers (*n* = 3) (for details see Table 1, for information about the caregivers see Table 2 and for information about the autistic children see Table 3).

**Table 1.** Participants per stakeholder group and location within Argentina.

	Buenos Aires	Rio Negro	Santa Cruz	La Rioja	Tierra del Fuego	Salta	Jujuy	Total *N*
Clinicians (psychiatrists, psychologists, nurses)	4	2	n/a	n/a	2	n/a	1	9
NGO representatives	3	n/a	n/a	n/a	n/a	n/a	n/a	3
Policy representatives	2	n/a	n/a	n/a	n/a	n/a	n/a	2
Teachers (special education teaching and regular schools)	n/a	3	n/a	n/a	n/a	n/a	n/a	3
Caregivers	7	2	1	1	2	1	1	15

n/a indicates no participants were recruited from this group or geographical location.

**Table 2.** Socio-demographic characteristics of caregivers.

**Caregivers**	
**Average age**	42.3 years
**Sex**	*N* (%)
Fathers	2 (13%)
Mothers	13 (87%)
**Level of education**	
Primary school	1 (6.6%)
High school	5 (33.3%)
Higher education	6 (40%)
No information provided	3 (20%)
**Marital status**	
Married or long-term partnership	11 (73.3%)
Single	4 (26.6%)
**Employment status**	
Full time work of self-employed	10 (66.6%)
Unemployed	3 (20%)
Retired	1 (6.6%)
No information provided	1 (6.6%)

**Table 3.** The age and diagnosis of the children.

Caregiver number	Child’s age	Child’s diagnosis
A1	2 years	ASD
A2	3 years	ASD
A3	16 years	ASD
A4	14 years	ASD
A5	13 years	ASD
A6	13 years	ASD
A7	13 years	ASD
A8	9 years	ASD
A9	15 years	ASD and ADHD
A10	12 years	ASD
A11	7 years	ASD
A12	4.5 years	ASD
A13	2 years	No formal diagnosis, suspected developmental delay
A14	4 years	ASD
A15	17 years	ASD
The average age of children:	9.6 years	

Three main themes were developed: Caregiver agency: from intuitive coping strategies to entrepreneurship; ‘I had to cut down on therapy’ – Economic instability and inequality affecting service access; and Equipping caregivers to be empowered. The main themes will be illustrated with direct quotes from participants. A full list of relevant quotes is included in Supplementary file 3.

### Caregiver agency: from intuitive coping strategies to entrepreneurship

Many caregivers explained that they saw themselves as the keys to supporting their child and added that it was a long journey to get to this point. Some mentioned struggling with deciding how to support their children because of their ongoing worries about the diagnosis, the future of the child and the feeling of helplessness. Many said that they experienced poor mental health at some point during their journey of supporting their child and had no support. Some mentioned grieving life opportunities they had before having an autistic child:

I stress about everything. I get upset with everything because I like to have things under control . . . my life was about planning: I get a degree and then I find a job, once I had the job I get married and have children, once I had children I travel . . . So, this [having an autistic child] takes me off my balance because I cannot plan anything . . . I stopped planning about work, about everything. I get angry because I get upset . . . (AC2, caregiver)

A few caregivers mentioned that they regularly organised mental health support groups for caregivers, facilitated by a psychologist. They said that these meetings provided a safe space for caregivers to talk about their own worries and experiences.

Many caregivers shared that as they had more access to information about autism and services, they started becoming advocates for autistic children and setting up initiatives solving problems they faced. Some caregivers spoke about transforming social spaces to be autism friendly. For example, a caregiver talked about how their caregiver association created a space for autistic children in a cinema to take a break from a movie if they feel overstimulated. Other caregivers spoke about a joint initiative with health professionals to create guidance on taking blood from autistic children:

I believe that it is quite distressing to get into the doctor’s office with children, specifically with autism or another kind of difficulty. What happens is more difficult to understand [for the child]. Since we had gone through that experience with my son’s blood draws, we thought . . . we need to do something. Nothing had ever been done. . . . [With] my son’s therapists . . . We made a protocol for the doctors in charge of the blood draws and for the parents: the social story would be a sequence of ready-made visual supports, real images that show from the moment the child gets into the laboratory until the moment he leaves . . . (AC3, caregiver)

There were a few caregivers who said that they were satisfied with the support they were already receiving. As they did not expect more support, they wished to use their energy to support others:

The truth is that for my family what we have is enough, I don’t need anything else. He [her autistic son] is older, very independent, he stays at home alone with his brother . . . I would like to find more participants for the [parent] association and to find a place for it where we can welcome parents and do everything we want in there, right? Because there are many things to do yet. (AC10, caregiver)

Many professionals thought that psychological empowerment of caregivers was the key to enabling them to support their families. However, a few raised concerns and suggested that there should be a clear division between the role of parents (caregiving) and the role of therapist. They worried that caregivers might spend too much time advocating for services and not spend enough time with their child:

To me it [empowerment] is a controversial point, okay? Because we can empower those parents, but making sure that they do not turn into therapists or professionals . . . I agree with empowering parents, but yet it seems more relevant to me the parent-child relationship at home than the relationship of child-treatment. (C1, NGO representative)

### ‘I had to cut down on therapy’ – economic instability and inequality affecting service access

Participants discussed in depth the barriers to achieving empowerment. They described that economic instability and poverty affected how caregivers could support their child. Caregivers mentioned the challenge of having to prioritise among various family needs. Many caregivers from low-resource backgrounds said that they worked temporary jobs. They explained that if they did not have enough earnings in a month, they had to reduce service usage. Some clinicians added that the attendance for caregiver interventions depended on whether the caregiver had a job in a given week. Some caregivers added that the COVID-19 pandemic increased their economic uncertainty:

I had to cut down my son’s therapy sessions . . . because of the pandemic my husband wasn’t working so we didn’t have enough money . . . the economic factor is a problem and I think it is the same for any person that depends on the public health system. (AC14, caregiver)

Both caregivers and professionals thought that inequities in accessing services and information impacted caregivers’ opportunities to support their child. Many health professionals thought that families in lower resource settings knew less about autism and what support was available in the first place. As an example of inequity, some caregivers mentioned that having contacts in health care helped them get an appointment with the right professionals. Others without these contacts may not access services or may have to wait substantially longer for an appointment. As the quote below illustrates, some professionals discussed the differences in living conditions and access to infrastructure as limiting factors to caregiver choices:

Here the social situation has extreme characteristics: there are extremely poor people, which here we call ‘people from the heights’ because they are in the mountains, they live in very rough weather conditions and . . . I was shocked when I arrived in here and started working as an occupational therapist . . . ‘Does he bath by himself? [I asked] – No, [said the caregiver] because we have a bucket, we don’t have a shower, I throw water on him with a bucket’ . . . or maybe ‘because we have soil floors and when it rains it gets wet’. So that was a reality that I had never experienced before, living and working in Buenos Aires. (D3, school teacher)

An example that both caregivers and professionals mentioned, that showed the intertwined challenges that inequalities produce, was obtaining a disability certificate for the child. Caregivers explained that having this certificate allows families to access certain interventions for autism for free. They added that the services covered depend on whether the family has health insurance – putting families in poverty at a disadvantage from the start. Caregivers highlighted that they found it challenging to access information about the certificate and stressed that obtaining the document was a lengthy process. Even when they had obtained the certificate, health insurance providers often tried to deny access to interventions. The result was that only the families who could afford seeking legal help could then go on and fight for the services they should have access to by law. A few caregivers who did not have health insurance explained that their only option was to pay for interventions out of pocket – which was often unaffordable. Professionals added that they often witnessed caregivers feeling hopeless about the challenges of dealing with insurance providers:

. . . it is quite common for them [health insurance providers] not to cover for anything until you ask for a Wit of Amparo [legal assistance] because it is the child’s right and it is mandatory for them to fulfil it. But [caregivers] have to fight a lot and this gets the families tired and it depends a lot on each parent’s personality to be able to fight. There are parents who get depressed because of this. (B1, health service provider)

Some clinicians in public hospitals said that single mothers living in *villas* (low-resource neighbourhoods of cities) are impacted by a combination of challenges, such as living in poverty, having no support network to fall back on and experiencing domestic violence. Other clinicians added that many single mothers struggled to work because of their care responsibilities. Caregiver participants who were single mothers talked about the difficult decision they were forced to make between supporting their child financially and having less or no income but be able to stay at home with the child:

In the region where we are, petrol is a prominent activity. The [studies] that I was doing was for working [in the petrol industry and earning a stable salary] . . . So, I considered whether I wanted as a mother that course or not, it was either economic welfare or supporting my child [and staying at home]. And now it is like I’m looking forward – I left the course on standby – to getting as much knowledge as I can about this topic [autism as part of a caregiver intervention] so tomorrow I can help other parents that are also going through this. (AC1, caregiver)

### Equipping caregivers to be empowered

Caregivers suggested that an important outcome of empowerment is to mitigate inequalities in service and information access. First, they thought that caregivers should be able to learn more about autism and their family’s rights. Some highlighted the importance of knowing whether the services they received are evidence-based. They suggested that training on critical thinking could help them navigate the overwhelming amount of inconsistent information available on the Internet. Others proposed building a centralised database of existing services, how they work and how families can access them:

In the beginning I would read everything I found. I looked up on the internet and I read, read, and read. Then, the neurologist told me not to because there were many articles unauthenticated on the internet. She told me to only pay attention to a particular organisation’s articles; she said ‘if it says the name of this organisation or if they recommend it read it, but nothing else’. So now I enter and check the author. (AC14, caregiver)

Second, caregivers explained that peer support groups can promote their empowerment and alleviate the impact of inequalities. For example, some caregivers mentioned that in the support groups they can learn about existing intervention approaches and hence make a better-informed choice for their child. Some caregivers argued that the online support groups set up during the COVID-19 pandemic were just as helpful in creating a sense of belonging as in-person meetings. Moreover, meeting online meant that those with less resources or living in a remote location could also participate. For these benefits, some caregivers suggested that the government should scale up online support groups across provinces where caregiver associations did not exist yet. Others highlighted that while caregiving was still considered to be a female responsibility in Argentina, more and more fathers started taking an active role in caretaking. They therefore suggested that support groups need to be set up where both fathers and mothers feel comfortable sharing their experiences:

We went to the countryside and there the men met each other, fathers, because it is always us, mothers, who take children to therapy. So, there they met, they did an asado [Argentinian grill as a social activity], and they saw how similar their children were and how similar they behaved. So, they were relieved, because fathers always have worries, but they don’t talk about them. (AC9, caregiver)

Third, many caregivers and professionals thought that caregiver-mediated interventions could increase caregiver empowerment. Many caregivers thought that knowledge exchange can increase their feeling of having just as much influence over their child’s improvement as professionals. Some professionals added that knowledge sharing can allow for continuity of support in-between intervention sessions at home – potentially improving intervention outcomes for the child. Others suggested that caregivers may feel overburdened by the expectation of having to learn what professionals may deliver in a health setting:

For example, [in an intervention] I would send videos with some ideas of how to play with their children and they [caregivers] answered with recordings of how they played, how the situation was, and then, together, we would analyse the interaction . . . you can work with many parents even without working with the child in the office. And there are parents who don’t have those [skills and resources] and you have to lead them and, sometimes, we need to consider to what extent we can ask them to do certain things because they might give up. (B2, health service provider)

Finally, both caregivers and professionals thought that caregiver initiatives could be even more successful if existing disability-friendly policies were also implemented in practice. Despite the favourable legal framework in Argentina supporting autistic individuals, all participants shared the view that what existed on paper often did not translate to the grassroots. Professionals added that autism was rarely prioritised among policymakers and that this meant that gaps in implementation remained unresolved:

In my experience policymakers are not interested in disability, it’s not on the agenda, of the 24 provinces only 5 have implemented the national autism law locally . . . For example, families should have access to inclusion specialists in the school but this is often non-existent in reality. This is called by lawyers mockingly the ‘rights on the paper level’. In order for this to change, the interest should be there from politicians. (E2, ministry & policy perspectives)

## Discussion

We investigated experiences with caregiver empowerment for families raising autistic children in Argentina. Across the themes we developed, both caregiver and professional participants discussed that caregivers’ ability to support their children was challenged by financial instability and pronounced socio-economic inequalities. Such challenges and how they impede service access are also well documented in previous work (Novick, 2017; Rubinstein et al., 2018). Moreover, previous research highlighted that the public health system in Argentina leaves a large part of the population uncovered (Novick, 2017). These unequal socio-economic conditions led to vastly different opportunities caregiver participants could choose from. This is in line with Sen’s capability approach suggesting that access to resources, and public goods depend on one’s socio-economic environment (Sen, 2014). Many caregivers discussed that lacking information about autism and DDs, and challenges with accessing support often made them feel disempowered. These concerns are also noted as an ongoing challenge across many Latin American countries (Montiel-Nava et al., 2020). Among caregiver participants, single mothers experienced further challenges: they had to make decisions such as whether to prioritise career development for an increased salary in the future, or spending more time with their child in the present. Some clinicians added that they were supporting single mothers who were victims of violence and who did not have a support network. Previous studies from a range of income settings also noted that single mothers often experience poverty, may lack social support and may have been victims of domestic violence (Levine, 2009; Mier y Terán Vásquez, 2021; Tekola et al., 2020). Domestic violence against women is common in most countries of Latin America, and there is a growing body of evidence looking at the experiences of mothers of children with developmental disabilities with domestic violence (Mier y Terán Vásquez, 2021). These combined challenges point to the importance of taking an intersectional approach when supporting families with autistic children (Ben-Moshe & Magaña, 2014) and create clear referral pathways, so that caregivers receive holistic support from managing their child’s condition to tackling socio-economic difficulties (Knifton & Inglis, 2020; Torres et al., 2018).

Overall, most caregivers thought that accessing information about their families’ rights would be a crucial first step in their empowerment journey. This rights-first approach is promoted by literature suggesting that caregivers should be key agents of change as compared to sole beneficiaries of services (Luttrell & Quiroz, 2007). Caregivers shared their journey of becoming advocates and social entrepreneurs. The empowerment journey was not without challenges: many mentioned having experienced mental health concerns. Some participants mentioned the example of a caregiver association organising caregiver well-being support in the form of a peer group. Previous literature has also noted that focusing on caregiver well-being in a group format can help increase caregivers’ sense of control over their children’s support (Ault et al., 2021; Papadopoulos et al., 2019).

Caregivers mentioned a wide range of initiatives they created from advocacy campaigns to widening access to education and employment for their children. However, many of these projects remained local solutions. An opportunity seems to arise here: systematic information could be collected by researchers about existing successful caregiver initiatives across the country so that they could then be scaled across provinces through the government. This could be a means towards bridging the gap in service access across urban and rural areas. Moreover, it would bring forward caregiver-led solutions that are more likely to be acceptable across communities in Argentina (Yardley et al., 2015).

Caregiver and professional participants mentioned three ways in which caregivers could be further equipped to be empowered. First, both caregivers and professionals discussed the empowering effect of caregiver support groups, as also highlighted in previous literature (Ault et al., 2021; Oh & Lee, 2009). Some informants mentioned that fathers were increasingly involved in supporting their child and that therefore support groups should be inclusive of the needs of both mothers and fathers. This is in line with the global trend of fathers raising their voice for their children (Cabrera et al., 2018; Rudelli et al., 2021). Second, caregivers and professionals highlighted that caregiver-mediated interventions co-designed by caregivers and clinicians could help caregiver empowerment and mitigate inequalities in service access (Rattazzi, 2014). Leveraging caregiver knowledge and professional expertise was highlighted as an advantage of these interventions, also described in previous literature (Rattazzi et al., 2020; Szlamka et al., 2022). Professionals added that caregiver programmes allow for the continuity of support at home, as noted in earlier work (Factor et al., 2019; Kasari et al., 2014). Some caregivers mentioned that they wished there was evidence available to them about the effectiveness of interventions. This suggests that researchers and practitioners can help caregivers by directly communicating research outcomes to them. Using evidence-based health communication principles can be a way to support this approach (Kreps, 2012). Participants in policymaking referred to the existing policies in Argentina that outline the rights of families with autistic children (Moldavsky et al., 2011; Palacios et al., 2020). They discussed that these policies are rarely implemented at the provincial level. Many believed that autism is not a priority for policymakers, a challenge described in literature across Latin America (Montiel-Nava et al., 2020). They suggested that system-level efforts are necessary to level some of the inequalities that cause barriers to service access at the first place, in line with the literature (Palacios et al., 2020).

Finally, participants spoke about increasing collaborative efforts among caregivers and professionals, acknowledging the formal expertise by professionals and the intuitive knowledge of caregivers. This approach has been described as essential in designing caregiver interventions and in implementing a participatory approach in health services (Fiest et al., 2018). In the case of a few professional participants, empowerment was seen as a concept that blurs the line between the responsibilities of caregivers and professionals. A few believed that empowerment might even impede the caregiver from supporting the child by focusing too heavily on interventions and advocacy. In caregiver-centred services, expected roles and outcomes should be discussed between professional bodies and caregiver associations. Existing work on participation and inclusion in research and service development can help guide these discussions (den Houting, 2021; Fetterman et al., 2014).

### Limitations

This study has various limitations. By focusing on caregiver perspectives, the voices of autistic individuals themselves were lacking in this work. We focused on caregiver empowerment as caregivers are the main advocates for support services in the early years of child development. Future studies should include the voices of autistic adults. These perspectives can add what autistic people wished their caregivers would have advocated for. Caregiver informants were almost entirely female and hence fathers’ voices are less represented. Some caregivers might have been afraid of participation due to stigma. Therefore, the voices of families in heavily stigmatised settings may also be underrepresented. Furthermore, we only managed to interview two policy representatives. More interviews might have resulted in the collection of further relevant data.

In terms of methodology, since all interviews took place online, some aspects of non-verbal communication may have been lost. It also means that potential informants who lacked access to Internet connection or a device for online calls could not participate. As the first author is not a native Spanish speaker, she may have misinterpreted some information shared by informants. However, she worked with a native speaker research assistant to transcribe and translate interviews. Furthermore, throughout the study, she worked closely with the second author who is a native speaker.

## Conclusion

Caregiver empowerment can be an important next step in further developing services and service access for families with young autistic children in Argentina. This study identified opportunities and existing initiatives for supporting caregiver empowerment within the existing health, social care and education system. Examples include supporting caregivers learning about their families’ rights, scaling peer support groups and allowing access to caregiver-mediated interventions. Caregiver empowerment can be promoted by the inclusion of families across all services and social spaces: by addressing the strong impact of socio-economic inequalities on the quality of life of families, focusing on caregiver mental health and further strengthening collaboration between professionals and caregivers.

## Supplemental Material

sj-docx-1-aut-10.1177_13623613241238254 – Supplemental material for Caregiver-centred empowerment for families raising autistic children: A qualitative case study from Argentina Supplemental material, sj-docx-1-aut-10.1177_13623613241238254 for Caregiver-centred empowerment for families raising autistic children: A qualitative case study from Argentina by Zsófia Szlamka, Cukier Sebastián, Charlotte Hanlon and Rosa A Hoekstra in Autism
