# Supplementary material for: Caregiver-centred empowerment for families raising autistic children: A qualitative case study from Argentina
Source: Autism. 2024 Mar 18;28(11):2768–78. doi: 10.1177/13623613241238254 (PMC11497749; doi:10.1177/13623613241238254)
Supplement: sj-docx-1-aut-10.1177_13623613241238254 – Supplemental material for Caregiver-centred empowerment for families raising autistic children: A qualitative case study from Argentina [file sj-docx-1-aut-10.1177_13623613241238254.docx]

# Supplementary files

These are the supplementary documents to the manuscript titled Caregiver-centred empowerment for families raising children with autism or other developmental disabilities: A qualitative case study from Argentina.

# Supplementary file 1.: Full topic guides

**Topic Guide for Caregivers in Argentina**

**Warm up questions (depending on the relationship with the caregiver):**

In this interview I’m interested in hearing about your child with a neurodevelopmental disorder, services that are available to support you; and what you think would be helpful for your family.

Can you tell me about your family in general? [Who do you live with?] How was your last week? [What happened to your child? What happened to other family members?  What was the best part of the week? What was the most difficult part of the week? Are these experiences regular?]

**Experiences and needs**

If you have any concerns about your daily life or your family, what do you do? Is there someone specific who you talk to? *[How/why did you choose this person?]*

Is there something else you wish you could do to handle these concerns? Is there someone else you wish you could talk to about your concerns? *[Would they be able to help? If so, in what way? Is there anyone who you think could help you in some way? In what way could they help?]*

Let’s now talk about having a child with a neurodevelopmental disorder. What are the key difficulties your child is experiencing?

If you have any concerns about your child, what do you do?

Who do you talk to? *[How do you decide what to do? What do you talk about with this person? (neighbours, friends, doctor, teacher, health worker, religious leader)]*

Do you have any concerns about your child now? *[If so, what are they?]*

Do you have any concerns about your child in the future? *[If so, what are they?]*

What do you do about these concerns? *[How do you try to overcome your concerns?]*

Can you tell me about things that you find important to provide your child or family with? *[Is there something you would like to be able to provide but you can not as for now? If so, can you tell me more about this? What would you like to have or provide to your child that you think would help your family?]*

Are you currently using services supporting your child, your family and/or yourself?

What are your experiences with these services? *[Is there anything that could support you further?]*

Have you used different services before? If so, what were your experiences? *[What made you change services?]*

In your experience, how does your community look at your child? Have you ever experienced something particularly positive or negative regarding how your community engages with your child and your family?

With the COVID-19 pandemic many of us have experienced changes in our life. How has COVID-19 impacted you and your family?*[And your child specifically; services available and the ways in which they are accessible?]*

**Society and rights**

To what extent do you feel you have enough information about your rights and your children’s rights for service development?

What are the most important rights for you? *[Tell me about your thoughts.  Who did you learn from about your rights?]*

Where did you get informed about your rights?

To what extent do you think there are services available to meet your rights? *[Tell me about a good example and a bad example.]*

Who do you think should know more about children with neurodevelopmental disorders? *[Can you give examples of an organisation or a person?]*

**Experiences with caregiver interventions**

How have you (as an individual, as a family) tried to overcome the challenges you face? *[What has helped you? What has got in the way?]*

Have you ever attended a programme specifically for parents who have a child with a neurodevelopmental disorder? Can you tell me about it? *[What was the programme about? How did you get to know the programme? Why did you attend? What was your expectation from it? How was your experience?]*

What is your experience with meeting parents who also have children with DD?

If you attended a caregiver intervention: Based on what your learnt during the programme, what do you think you would still need to overcome the challenges you are facing?

**To explore gender-related themes:**

You mentioned your [partner/other significant caregiver] earlier. How does s/he relate to your child with DD?

Does s/he take on care duties? If so, what are they? How do you make decisions about who takes on what duty?

Do you talk about your child with your [other significant caregiver]? *[How do those conversations go?]*

How do other family members relate to your child?

Do you talk about your child with other family members? *[How do those conversations go?]*

*For those who have no experience with other parents’ groups/interventions:*

What do you think could help you to best support your family and child?

Can you tell me about things that you find important to support your child or family with? Is there an area where you would like to offer support but you can not as for now? *[If so, can you tell me more about this?]*

Have you heard of anything (from other families, neighbours, friends) that exists in other towns, provinces, in Buenos Aires or other countries that you think could help you?

**Topic guide: NGO, social worker and academic representatives**

**Meeting the needs**

Can you tell me briefly how you started working in your area of expertise?

In what ways have you come across families with children with neurodevelopmental disorders? *[What did you do in this situation? What did your organisation do?]*

In your experience, what do families raising a child with a neurodevelopmental disorder need? *[How did you get to know this? How do you think these needs can be met?  How should this be done in practice?]*

**Empowerment**

What do you think families with a DD expect from your organisation?

What do *you* think is the role of your organisation in supporting families with DDs? *[Can you give examples of what works well and what doesn’t work so well?*

Have you ever come across a difference in expectations across families and your organisation?

When talking to different organisations, the concept of empowerment of families came up on occasion. However, what I found was that different people mean different things by empowerment. What does empowerment mean to you (in the context of working with families with a child with DD)? What would it mean for a family to be empowered? *[To what extent is empowerment a goal in your work?]*

What do you think would be a good outcome of empowerment? *[What would you consider as successful? What would you consider as unsuccessful? Who do you think is responsible for making sure these happen? Who should initiate these practices?]*

Are there any techniques that work well to achieve these goals? Are there techniques that do not work well? How do you know?

What would you prioritise as a resource to be made available for these families?

With the COVID-19 pandemic many of us have experienced changes in our life. How has COVID-19 impacted your work?*[And services available and the ways in which they are accessible?]*

Can empowerment be unsuccessful? If so, what happens?

**Supporting intervention providers and caregivers**

Can you tell me your experience about working together with caregivers? *[What is the role of those working in your organisation? What is the role of the caregiver? What is the caregiver’s contribution?]*

How do caregivers get what they need from your programme? *[Is there anything that interferes with that?]*

Do you have any good practices of supporting that those working in your organisation and caregivers remain involved in the intervention?

Can you tell me about any stakeholder groups you are working with regularly or on occasion? *[What is it like to work with different professionals and different groups? Is there something that works particularly well with any of these groups? Is there something you find challenging when working with them?]*

**Experiences with caregiver interventions**

Let’s now talk about interventions. In your experience with available services, who is the most common beneficiary? [Are services directed to the child or to the family?]

Have you come across any caregiver interventions in your work? Can you tell me about your experience with them?

What would help in implementing caregiver-mediated interventions in your area? *[Is there anything that hinders such development? What could be done about it?]*

*For those working with caregiver programmes:*

Let’s talk about the intervention you are working with. What are your experiences with supporting caregivers and families attend the intervention? *[Are there any key challenges of engaging caregivers? How did you choose this intervention? How would you summarise the key goal this intervention is hoping to achieve?]*

How do you think caregivers should be approached regarding the participation in the intervention? *[Do you have any practices that worked well or didn’t work very well in this regard?]*

In your experience, what’s the view of parents on an intervention in which they are required to play an active role?

What do you think about the participation of caregivers and families from lower socio-economic backgrounds or of those who received little education?

Did you find any approaches or practices that work well to engage caregivers from a variety of backgrounds? *[Did you find approaches that didn’t work well in this regard?]*

What do you think about paying caregivers to attend the intervention?

To what extent do you think the caregiver programme meets the most important needs of caregivers?

**Training and teaching (in case if the participant has relevant experience)**

In the intervention you are working with, there may be concepts introduced that are unfamiliar to the target audience. Have you come across such a situation before? *[If so, what was your experience?]*

Were there any concepts or teaching methods that caregivers found difficult to understand? *[Did you find any solutions to this? Did you find any techniques that worked particularly well explaining new concepts to caregivers? Did you find any techniques that proved to be unhelpful?]*

In your experience, was there something caregivers didn’t find relevant? *[Can you give examples?]*

To what extent are you able to use the experiences of caregivers to develop existing programmes?

Do you have any good practices of how to help caregivers attend to the programme?

Have you come across examples whereby the caregivers themselves shared relevant experiences that were helpful to others in the group?

In your experience, was there something caregivers didn’t find relevant? *[Can you give examples?]*

**Pricing of interventions and stakeholder groups**

Let’s now talk about how interventions are financed. Some people argue such programmes should be free while others state participants should pay a fee to attend. *[What do you think about this? Can you explain why? How do you think the price may relate to how the intervention works?]*

**Topic guide: Representatives of local health, education and social care authorities**

I would like to talk to you today about your experiences with families raising a child with a developmental disorder, as well as about the ways in which they may be empowered.

**Meeting the needs**

What type of activities do you know of for people with disabilities in this district? *[How do you know about these activities? How are they relating to your sector and profession?]*

**Empowerment and service development**

What does empowerment mean in your understanding? *[What do you think the goal of empowerment is? What are the means of empowerment? Are there techniques that do not work well? How do you think this intervention is achieving these goals?]*

What do you think families with a DD expect from your institution? *[To what extent do you think you meet these expectations?]*

What do you think is the role of your institution in supporting families with DDs? *[Can you give examples of what works well and what doesn’t work so well? What is the role of other institutions you are working with?]*

With the COVID-19 pandemic many of us have experienced changes in our life. How has COVID-19 impacted your work?*[And services available and the ways in which they are accessible?]*

**Pricing of interventions and stakeholder groups**

Let’s now talk about how interventions are financed. Some people argue such programmes should be free while others state participants should pay a fee to attend. What do you think about this? *[Can you explain why? How do you think the price may relate to how the intervention works?]*

Can you tell me about any stakeholder groups you are working with regularly or on occasion? *[What is it like to work with different professionals and different groups? Is there something that works particularly well with any of these groups? Is there something you find challenging when working with them?]*

*For those who have more experience with caregiver interventions:*

Let’s talk about interventions in general. In what ways do you get informed about what services are available and what could be made available for families in your area?

What would you like to know about a programme before you decide whether to give your support?

How do you go about choosing interventions to support?

What are your expectations from these interventions?

Once a programme is supported, how much do you remain involved in its implementation?

Which stakeholder groups would you like to see in the development of these programmes?

What do you think about getting communities from a variety of backgrounds attend these programmes?

What do you think about paying or reimbursing participants to attend such interventions?

What questions would you ask once the intervention programme is finished?

To what extent do you find programme sustainability relevant?

What are the ways in which programmes can work more effectively with the government sector?

# Supplementary file 2.: The final codebook

| Name | Description |
| --- | --- |
| Caregivers initiating change |  |
| Caregiver-initiated interventions |  |
| Autism friendly blood draw |  |
| Autism friendly cinema |  |
| Awareness raising |  |
| Campaigning against noise |  |
| Caregivers as interventionists |  |
| Creating a community for children with NDDs |  |
| Early motor stimulation project |  |
| Initiating legal changes |  |
| Psychological support for caregivers |  |
| Running a caregiver association |  |
| Systematic assessment of child development |  |
| Taking control over the child's improvement |  |
| The goal is independent living for the child |  |
| The mental health of caregivers | professionals are supporting caregiver mental health, caregivers are often fatigued and therefore it is harder to work with them in an intervention |
| Professionals and family working together | coordinating meetings can be challenging |
| Caregiver and professional experiences with existing caregiver interventions | by puberty caregivers become exhausted of interventions, caregivers paid by health insurance can also become exhausted |
| Parent groups are needeed for better services |  |
| Managing caregiver expectations |  |
| Caregivers are ready to do everything for the child |  |
| How caregivers are viewed by professionals |  |
| Caregivers are overly professionalised |  |
| Caregivers come to consultation to seek answers |  |
| What caregivers think interventions can achieve |  |
| What to say about prognosis |  |
| Relying on caregivers' intuitive expertise |  |
| The caregiver perspective on empowerment |  |
| Drawbacks to empowerment |  |
| Empowerment as skills and capabilities |  |
| No need for anything else |  |
| Support groups |  |
| Lacking parent groups in certain provinces |  |
| Online groups help caregivers claim their rights |  |
| Other parents can give the psychological support needed after the diagnosis |  |
| Support groups could be supported by government |  |
| The children should be empowered |  |
| The first step is for caregivers to know their rights |  |
| Discussions about inclusion in Argentina |  |
| Beliefs about autism |  |
| Autism is trendy on a worldwide level |  |
| Stigma and awareness |  |
| Awareness depends on the location | in some provinces it is lacking |
| Awareness is increasing and attitudes are changing towards autism |  |
| Awareness needed about other NDDs |  |
| The motivation to work with NDDs |  |
| Views on what causes autism |  |
| Views on what is or is not available abroad |  |
| Experiences with education from an inclusion perspective |  |
| Experiences with different types of schools |  |
| Fighting for support with education |  |
| Inclusion requires interdisciplinary work |  |
| Inclusion service in rural areas |  |
| More teacher training is needed for inclusion | experiences with support teachers, teachers should change their attitude towards autism |
| Nothing available after primary school |  |
| Special education specialists as responsible for inclusion of autism |  |
| When is it helpful for a child with autism to go to the regular school |  |
| Caregivers want integrated education |  |
| If the child receives extensive therapy outside the school environment the child can get exhausted |  |
| Inclusion should start in kindergarden where there is more neuroplasticity |  |
| Inclusive education teaches other children about autism |  |
| The complementary inclusion strategy | one reason is to address heterogeneity in autism |
| What and how to achieve with inclusion |  |
| Current challenges with inclusion |  |
| Attitudes need to change regarding who should be adapting in the inclusion process |  |
| Inclusion means focusing on the individual strength of the child |  |
| Outlier cases |  |
| Service access in Argentina |  |
| Multi-sectoral work is needed so that support services are not isolated |  |
| Developing support that is freely accessible |  |
| Diagnostic challenges |  |
| It is unclear which sector should provide services for autism |  |
| Some professionals only started learning about autism recently |  |
| Strong psychoanalytical approach in Argentina |  |
| Support depends on individual leaders |  |
| Service access differs by location |  |
| Bringing services to a new region |  |
| Caregivers travel to the capital to have services |  |
| Knowing someone in the system helps in service access |  |
| Regional cities have services while rural areas are lacking access |  |
| Service access differs based on having knowledge and information |  |
| Service access differs by access to resources |  |
| The policy to implementation gap | great regulations exist but they do not take place in practice |
| Certain good practices are not picked up by policy |  |
| Disagreemens around health insurance to get treatment | caregivers fight to get access, professionals are paid only a low fee and therefore they hardly get by |
| NDDs are not a priority |  |
| What is needed for the future |  |
| Advocacy for inclusion, awareness and closing the policy to implementation gap |  |
| Caregiver interventions and caregiver involvement in therapy |  |
| Database with organised information on services |  |
| Early intervention for more children |  |
| Engaging social centres for early diagnosis and intervention |  |
| Evidence is needed about interventions and it has to be communicated to caregivers |  |
| Increasing access to social spaces |  |
| Locally available funding for disabilities |  |
| Making caregiver groups more organised |  |
| More professionals |  |
| It is professionals who have to adapt to caregiver needs |  |
| More interdisciplinary approach |  |
| Psychological support to caregivers |  |
| Services for teenage and adult years |  |
| Teacher training on autism |  |
| More breaks during the classes |  |
| Widen service access for example in mountainous areas |  |
| Supporting a child with autism in Argentina |  |
| Gender perspectives in caregiving | mothers and fathers collaborate, divorce may happen |
| Domestic violence |  |
| Examples of mothers and fathers working together |  |
| Experiences of single mothers |  |
| The father's perspective |  |
| The impact of COVID-19 on autism services | COVID can be a good excuse not to offer services to difficult cases |
| Impact on professionals |  |
| Impact on the children |  |
| Professionals and caregivers communicating |  |
| Online tools helps multisectoral work | internet access means more information to caregivers |
| The impact on caregivers |  |
| The impact of social determinants and poverty | a caregiver may have to decide between working and therefore economic welfare or staying at home and supporting the child |
| Priorities when raising many children |  |
| Social determinants impacting the family |  |
| The impact of poverty on service access | poorer families do not have access to services following diagnosis, families in poverty get services later and that goes against early intervention, relying on primary health care for early diagnosis in poorer settings |
| The journey of finding support |  |
| Caregiver journey to diagnosis |  |
| Having to explain autism in the education space |  |
| How family members may view autism differently | autism as something natural or a disease |
| How to share the diagnosis with others |  |
| Negative experiences with diagnostic services |  |
| What happens after the diagnosis |  |
| Caregivers sending the child to any therapy available | diet therapy, music therapy, horse riding, those who can afford it |
| Caregivers worry about the future skills of the child |  |
| Experiences with teenage years |  |
| Needing legal support to access services |  |
| Talking about autism with the child on the spectrum |  |

# Supplementary file 3. The list of the most relevant quotes

| **Theme** | **Participant** | **Quote** |
| --- | --- | --- |
| Equipping caregivers to be empowered | *B9, health service provider* | *“In fact, there are so many [caregiver associations] that I think it has turned into a problem. They are a lot and instead of making the energy and resources converge they compete with each other and no one gets anything. It is poorly organized…They spend time criticising professionals, paying attention to what they earn. They spend more time thinking about that than calling professionals to do one thing or the other. We are not really in touch…”* |
| I had to cut down on therapy – Economic instability and inequality affecting service access | *AC11, caregiver* | *“Overall he has worked on interaction with the speech therapists, with the psychologist. It did work. There were days in which we had to interrupt the session because he would get tired. To have to spend such a time in front of a phone…. That is difficult for us, adults; we are tired of the number of calls we have. How do you sit him forty minutes in front of a phone? But, anyways, there were therapies that he completed happily, and he made a lot of progress in many aspects. However, sometimes we had to finish the calls earlier.”* |
| Equipping caregivers to be empowered | *B9, health service provider* | *“Overall because they [caregivers] have that perspective “– Is it [caregiver intervention] for my son? –No, it is for you, mother” and therefore the excuses come up. Excuses and reality, because it [the intervention] is in the mornings so some mums have other children – “who would take care of them?”, some others have to work.”* |
| Equipping caregivers to be empowered | *AC8, caregiver* | *“I learnt that … moment of joint attention that we need to embrace to work on new things. It was very productive. Even when I used to do those things with him [my son], I didn’t know how to conceptualize it. Yes, because they explained it to me... “How do you connect with your son?” If someone had asked me that, I would have said that I am very affective and I really like to play with him, but I hadn’t known how to explain that, how to say it until they explained it and I thought “Oh this is what I am doing, this is it, they are explaining it to me, now I can explain what it is”* |
| I had to cut down on therapy – Economic instability and inequality affecting service access | *B7, health service provider* | *“Yes, there are a large number of families like that [that fall apart after receiving a diagnosis of a DD]… I don’t know if it is the most usual thing though, we should do some statistics. And sometimes they have another support role –which might be an uncle, a grandfather, a cousin– that is not the father figure but it is a masculine figure. And then there are other cases in which the support role is played by feminine figures… Or in other cases the mother has died and the father is in charge. We have had cases like those.”* |
| Equipping caregivers to be empowered | *AC8, caregiver* | *“I present him the stimuli but I don’t expect anything to happen quickly…I know that something is happening in his brain, in his body, but I don’t expect him to give me anything, at least while they are children…After a while you can ask for things in return but you shouldn’t get obsessed with your own expectations or what you wanted to happen.”* |
| Equipping caregivers to be empowered | *B3, health service provider* | *“It is not to criticise psychoanalysis but to understand that there are things that have changed and that as time goes by there are theories that are marginalized…or ways of intervention or ideas with which parents feel bad or guilty about what happens to their children, because it was thought that because they did something someway or because they hadn’t been able to interact with their children in a certain way, their children has these developmental issues.”* |
| Equipping caregivers to be empowered | *B1, health service provider* | *“One thing that occurred during pandemic, is that we created Whatsapp groups between the therapeutic team and parents…in these Whatsapp groups feedback and participation have been more frequent, I think.”* |
| Caregiver agency: from intuitive coping strategies to entrepreneurship | *AC8, caregiver* | *“I had to come and go many times [to the health insurance company], many times they answered “no”... but insisting was more powerful because when they said “no”, I will find the way to get a “yes”, because I had the law by my side, so I read articles, I started to know the law, because if you don’t know it you cannot ask for anything…I made myself a notebook and started controlling all the details …I made on my own my child’s medical record, to know what I needed…so I could have an organized record to take to the health insurance and so that they couldn’t say “no” for answer. That took me years...”* |
| Caregiver agency: from intuitive coping strategies to entrepreneurship | *AC2, caregiver* | *“Look, when I got the diagnosis [for my son], my mum sent me... My mum has a friend near here, from the same area, who has a daughter with autism, so my mum gave me her contact so I could talk to her, mainly to ask her about professionals around our neighbourhood. It was very good to me to speak with her, because she is a teacher and she had studied everything related with laws. And she told me that to know your rights on this topic is the most important thing in order to defend yourself.”* |
| Caregiver agency: from intuitive coping strategies to entrepreneurship | *AC1, caregiver* | *“My biggest dream or my biggest aim is to reach those parents that maybe feel too small and to show them that there are more people that maybe, because of lack of knowledge, don’t get involved or don’t learn about it.”* |
| Caregiver agency: from intuitive coping strategies to entrepreneurship | *AC10, caregiver* | *“If you get the [disability] certificate, you gain legal support to make use of any service you need, but in this country that right is not fulfilled…the truth is that some parents like us have resources and know that they can claim for their rights but for other parents that don’t have those resources it is rough.”* |
| Caregiver agency: from intuitive coping strategies to entrepreneurship | *AC9, caregiver* | *“We choose the therapy; we decide if meets our family’s needs and desires. We set goals for the future three or four months and we try to achieve them and if we don’t achieve those goals, we change either the therapists or our attitude towards the therapy. That took ages.”* |
| Equipping caregivers to be empowered | *AC1, caregiver* | *“And with the group of parents with who we share a WhatsApp group... It is impressive how it changes your mind, because before is like you were there in the emptiness of “What do I do?”, “What do I not do?”, “How do I continue?”, “How do I not continue?” On the contrary, today I have that group of parents as a tool, they tell you tips like “this worked for me”, “that worked for me”, we help each other. On the contrary, in my city I tried to communicate with other parents that had kids in the same condition, but it was like... they didn’t have the pedagogy or the tips or the support that the people from outside the city or the province had. I believe that in the region of Buenos Aires there are a lot of pedagogy, there are a lot of foundations, and that changes everything and it is… it is very sad that in my city it is like the autism is a taboo, because there is no awareness, because no one talks about it.”* |
| Equipping caregivers to be empowered | *B3, health service provider* | *“So, if we don’t train parents… the chance of making some progress is reduced to a minimum. If you blame parents for the condition they have, for the way they are, the parents feel terrible and they don’t make any progress. So, the treatment is with the parents and not making them feel guilty about what happens to their children. Psychoanalysis... um... well... The work that has been carried out by psychoanalysis on children with autism didn’t meet many results in terms of development...”* |
| Equipping caregivers to be empowered | *AC1, caregiver* | *“The most fundamental thing is for me to acquire as much knowledge as I can, so I will be able to give him [my child with DD] a support network; and for him to know that his mum will be always there for him as long as he needs her and, also, for him to understands that it is not him who has to get used to the world and has to fit in, on the contrary, he has to get out to the world the way he is and if people don’t like it, they are the ones who would have to fit in in his world.”* |
| Caregiver agency: from intuitive coping strategies to entrepreneurship | *B2, health service provider* | *“They invite us very frequently to give talks in schools and all of them are about autism. It is something that is appealing to people right now, which is a good thing, but there are also a lot of other things that we need to discuss around neurodevelopmental disorders, disabilities, different needs.”* |
| Equipping caregivers to be empowered | *E1, ministry&policy perspectives* | *“I think that many of us, who work in health and mental health and have a social vision of it, have the dream of offering high quality services to people in poverty conditions. And that is why I have always worked for the government.”* |
| Caregiver agency: from intuitive coping strategies to entrepreneurship | *AC3, caregiver* | *“Here in Argentina, I don’t know about other countries, is there research on…the families that had, I don’t know, occupational therapy, educational psychology…and what effects did it have? They give you the pack of therapies and that’s it, there is no research. And well it would be important to carry out research to see what is more effective, if it is the psychological approach or the biological approach, or both, do you know what I mean? As you can see, I’m a very curious mother.”* |
| Equipping caregivers to be empowered | *AC4, caregiver* | *“I will tell you about this pupil…at school. He has autism and he has a very low level but family want him to go to ordinary school and that’s a right that cannot be denied, even if people believe “he can barely read”. He is at his second year of high school and the difference with the class is quite big but the parents want that and it is his right. I don’t know if he benefits from that, but that’s a different thing.”* |
| Equipping caregivers to be empowered | *B6, health service provider* | *“They [caregivers] have power over the child – “power”, in fact they accompany him– but not over the professional. “I want him to speak” [says the caregiver] but he is not ready to speak, “so then we leave” [the intervention], [and I saw[ well, leave. So, we try to combine the parent’s desires with the professionals’. You try to prepare the parents, to tell them about the aims, about how development is...”* |
| Equipping caregivers to be empowered | *D1, teacher* | *“There are children who have a lot of academic skills, a lot of cognitive capacity, and there are children that have not. At that point we start looking for different strategies…At one time we implemented a double schooling strategy, the kid would go to an ordinary school twice or three times a week and twice a week to a special school, and he would receive special support.”* |
| Caregiver agency: from intuitive coping strategies to entrepreneurship | AC9, caregiver | “...we noticed there that there were different [intervention] approaches: cognitive behavioural therapy, Floortime…So, we thought it was a good idea for us, as a group of parents, to call for professionals that are involved with these approaches…for parents to choose the suitable treatment for their children based on their needs.” |
| Equipping caregivers to be empowered | B7, health service provider | “We always have to deal with that horrible thing of communicating to the parents the long-term prognostics when it is the case of a serious chronic condition like developmental ones in which you know that they would need lifelong assistance with the ups and downs of the case…Sometimes you can and sometimes you cannot, but I do think it is a way of helping outside the hospital…they need to know how to support them and how to provide them with the best environment possible.” |
| Equipping caregivers to be empowered | D2, school teacher | “We share WhatsApp groups, we carry out online meetings, that before —in person— were very difficult to carry out. So, now, the feedback is more frequent between the therapeutic teams and the school teams and this is very important because we contribute to each other’s job and we think together.” |
